# High Prevalence of HIV-Related Cryptococcosis and Increased Resistance to Fluconazole of the *Cryptococcus neoformans* Complex in Jiangxi Province, South Central China

**DOI:** 10.3389/fcimb.2021.723251

**Published:** 2021-11-01

**Authors:** Chunxi Yang, Zeyuan Bian, Oliver Blechert, Fengyi Deng, Hui Chen, Yueting Li, Yunhong Yang, Min Chen, Ping Zhan

**Affiliations:** ^1^Jiangxi Provincial People’s Hospital Affiliated to Nanchang University, Nanchang, China; ^2^Jiangxi Provincial Chest Hospital, Nanchang, China; ^3^Department of Dermatology, Shanghai Key Laboratory of Molecular Medical Mycology, Changzheng Hospital, Shanghai, China

**Keywords:** *Cryptococcus neoformans/Cryptococcus gattii* complex, multilocus sequence typing, antifungal susceptibility, resistance, fluconazole

## Abstract

**Background:**

Cryptococcosis is caused by a fungi of the *Cryptococcus neoformans/Cryptococcus gattii* complex and is a severe concern for public health worldwide. *C. neoformans* species are globally distributed, and *C. gattii* species are mostly found in America, Australia, and Sub-Saharan Africa. *Cryptococcus* usually infects an immunocompromised population; however, the majority of cryptococcosis in China has been reported in patients without any recognizable immunosuppression, i.e., HIV infection. To date, very few studies investigated this disease in South Central China.

**Methods:**

The present study recruited 230 clinically suspected cryptococcosis cases in the last 5 years at two hospitals in Jiangxi Province, South Central China. All isolated strains were subjected to multilocus sequence typing (MLST) and phylogenetic analysis. Serotype and mating type were assessed by PCR, *in vitro* antifungal susceptibility was assessed by the CLSI-M27-A3 protocol.

**Results:**

A total of 230 patients were identified as infected by *C. neoformans*, including 12 cases with *Talaromyces marneffei* coinfection. All seven MLST markers were successfully amplified and used to identify the ST genotype in 199 strains. *C. gattii* strains were not detected. In contrast to previous studies, 59.3% of the patients had an immunocompromised status, and 61.9% of these patients were infected with HIV. All isolates manifested serotype A and mating type α. The ST5 genotype was common (89.5%) in the Jiangxi region, and three novel genotypes (ST656, ST657, and ST658 in six isolates) were detected in the present study. A total of 86 of the isolates (43.2%) were not sensitive to fluconazole at a MIC_50_ ≥ 8 μg/ml, most of the isolates were resistant to amphotericin B, and nearly all isolates were resistant to itraconazole and posaconazole. Resistances to 5-Flucytosine and voriconazole were very rare.

**Conclusions:**

The results of the present study indicated that C. *neoformans* is the predominant species for cryptococcosis in Jiangxi Province, and a large proportion of the strains were not sensitive to fluconazole, which may be related to treatment failure and relapse. A high percentage of HIV-related C. *neoformans* infections was reported in Jiangxi, supporting a previous hypothesis that cryptococcosis is more frequent among the HIV-infected population in China. Continuous monitoring of species distribution and antifungal sensitivity is important for the investigation of this severe disease in the Jiangxi region.

## Introduction

Cryptococcosis is an invasive fungal infection with high morbidity and mortality. *Cryptococcus neoformans* and *C. gattii* are the main etiological agents of the disease. The pathogens are present in the environment in diverse ecological niches, e.g., eucalyptus trees and the feces of pigeons (Ellis and Pfeiffer, 1990; May et al., 2016).

Based on the pathogenicity and molecular biological characteristics, *C. neoformans*/*C. gattii* is divided in seven distinct taxa (Hagen et al., 2015). The taxon *C. neoformans* (former called *Cryptococcus neoformans* var. *grubii*) has a wide distribution and is the main pathogen of cryptococcosis in China. Strains of this taxon can be identified by PCR-fingerprinting and DNA sequencing (molecular type: VNI, VNII). Strains of *C. neoformans* have the serotype A (Meyer et al., 2003). *C. deneoformans* (synonymy *C. neoformans* var. *neoformans*) is characterized by the molecular type VNIV and having the serotype D. It has a wide distribution but is most commonly found in Europe. *Cryptococcus gatti* s.l. (*sensu lato*, in a broader sense) is now divided in five species: *C. gattii* (with the molecular type VGI), *C. bacillisporus* (VGIII), *C. deuterogattii* (VGII), and *C. tetragattii* (VGIV). These species are characterized by the serotypes B and C. *Cryptococcus gatti* s.l. has a worldwide distribution with hotspots in America, Australia, and Sub-Saharan Africa.

*Cryptococcus* is mostly found in the haploid form, growing as a yeast, and reproducing asexually; but they are able to mate and have the ability of sexual recombination. Even an interspecific hybridization between *C. neoformans*, *C. deneoformans*, and also *C. gatti* s.l. have been found (Hagen et al., 2015).

Spores or yeast cells of *Cryptococcus* are inhaled from the environment by humans and enter through the respiratory tract (Velagapudi et al., 2009). In immunocompetent hosts, the fungus normally is stopped by the immune system, even though the pathogen sometimes causes a latent and symptomatic infection (Qu et al., 2020). In immunocompromised hosts, *Cryptococcus* can cause pneumonia and can disseminate to other tissues, mainly to the central nervous system (May et al., 2016). In more than 80% of clinical cases with cryptococcal infections, cryptococcal meningitis were diagnosed (Rajasingham et al., 2017). Although the absolute number of cryptococcal deaths has decreased since 2008, the proportion of AIDS-related mortality remains high. An estimated 15% of cryptococcal meningitis are the cause of AIDS-related deaths (Rajasingham et al., 2017).

Although most fatal infections occur in southern Saharan Africa, an increasing number of cases have been reported in other regions, including China (Yuchong et al., 2012). Interestingly, the majority of cryptococcosis in China was reported in non-HIV patients (>80%), and infections were also reported from immunocompetent hosts (Fang et al., 2015; Zhou et al., 2020). A systematic literature review by Zhou et al. noted that cryptococcal meningitis occurred in China in immunocompetent individuals almost twice as often as in patients with AIDS (Zhou et al., 2020). Jiangxi Province is a large agricultural province in South Central China with a population of more than 45 million residents. The subtropical and humid climate is favorable for *C. neoformans* (Li et al., 2012). Occurrence of cryptococcal infections continues to increase; however, large-scale epidemiological data on cryptococcosis from this region are very limited (Chen et al., 2018). The two most important nationwide studies, reviewing the epidemiology of *Cryptococcus* in China, reported the retrieval of only very few strains from Jiangxi Province (Chen et al., 2008; Fang et al., 2015).

Only the study by Chen et al. (2018), about the epidemiology of cryptococcosis in Jiangxi Province, systematically analyzed the cryptococcosis epidemiology in this province, on the basis of 86 cases (Chen et al., 2018). The study included *Cryptococcus* cases occurring in a period of two years. Notably, all *Cryptococcus* strains were sensitive to routine antifungal agents: flucytosine, amphotericin B, itraconazole, voriconazole, and fluconazole. According to the Invasive Fungal Surveillance Net (CHIF-NET), resistance to fluconazole occurred in about 10% of the strains analyzed in China (Xiao et al., 2018). Thus, we increased the number of analyzed *Cryptococcus* strains collected in Jiangxi Province to obtain a more representative database on fluconazole resistance.

All *Cryptococcus* strains isolated in Jiangxi Province belong to the *Cryptococcus neoformans/Cryptococcus gattii* complex. The study of Chen et al. identified all 86 isolates as *C. neoformans* serotype A and mating type α (Chen et al., 2018). Recently, Cao et al. reported emerging *C. gattii s.l.* infections in Guangxi Province, south of Jiangxi Province (Huang et al., 2020). By increasing the number of *Cryptococcus* isolates, we aimed to provide a more accurate description of *Cryptococcus* diversity in Jiangxi Province.

Reports of *C. neoformans* meningitis (CM) were almost exclusively from patients of the hospitals associated with Chinese universities, whereas most Chinese HIV-infected patients are treated in specialized infectious disease hospitals. Thus, Chen et al. suggested that CM cases in the HIV-infected population in China may have been severely underreported (Chen et al., 2020). Additionally, Chen et al. (2018) reported that 40% of *Cryptococcus* infections were HIV-related.

Therefore, to obtain additional information about the species distribution in Jiangxi Province, molecular epidemiology, and antifungal-drug susceptibility, we conducted a two-center retrospective study including 230 clinically diagnosed cryptococcosis cases, which were all recorded in the last 5 years. The patients originated from nearly all regions of Jiangxi Province; a combination of MLST genotype analysis and *in vitro* antifungal tests was used for serotype and mating type determination. The aims of the present study were as follows: (1) to describe the species distribution and molecular epidemiology of clinical *Cryptococcus* in Jiangxi Province; (2) to explore the *in vitro* antifungal profiles of *Cryptococcus*, especially potential resistance to fluconazole; and (3) to describe the serotype and mating type loci in the clinical strains in South China.

## Materials and Methods

### Study Population and Geographic Region

The present retrospective study was conducted at two high level general hospitals in Jiangxi Province, Jiangxi Provincial Chest Hospital and Jiangxi People’s Provincial Hospital. The data were retrieved from the medical records stored at the hospitals, and all Jiangxi native patients diagnosed with *Cryptococcus* infections from July 2015 to June 2020 were recruited. Approval for the present study was granted by the Ethics Committee of Jiangxi People’s Provincial Hospital affiliated with Nanchang University.

**Figure 1:**
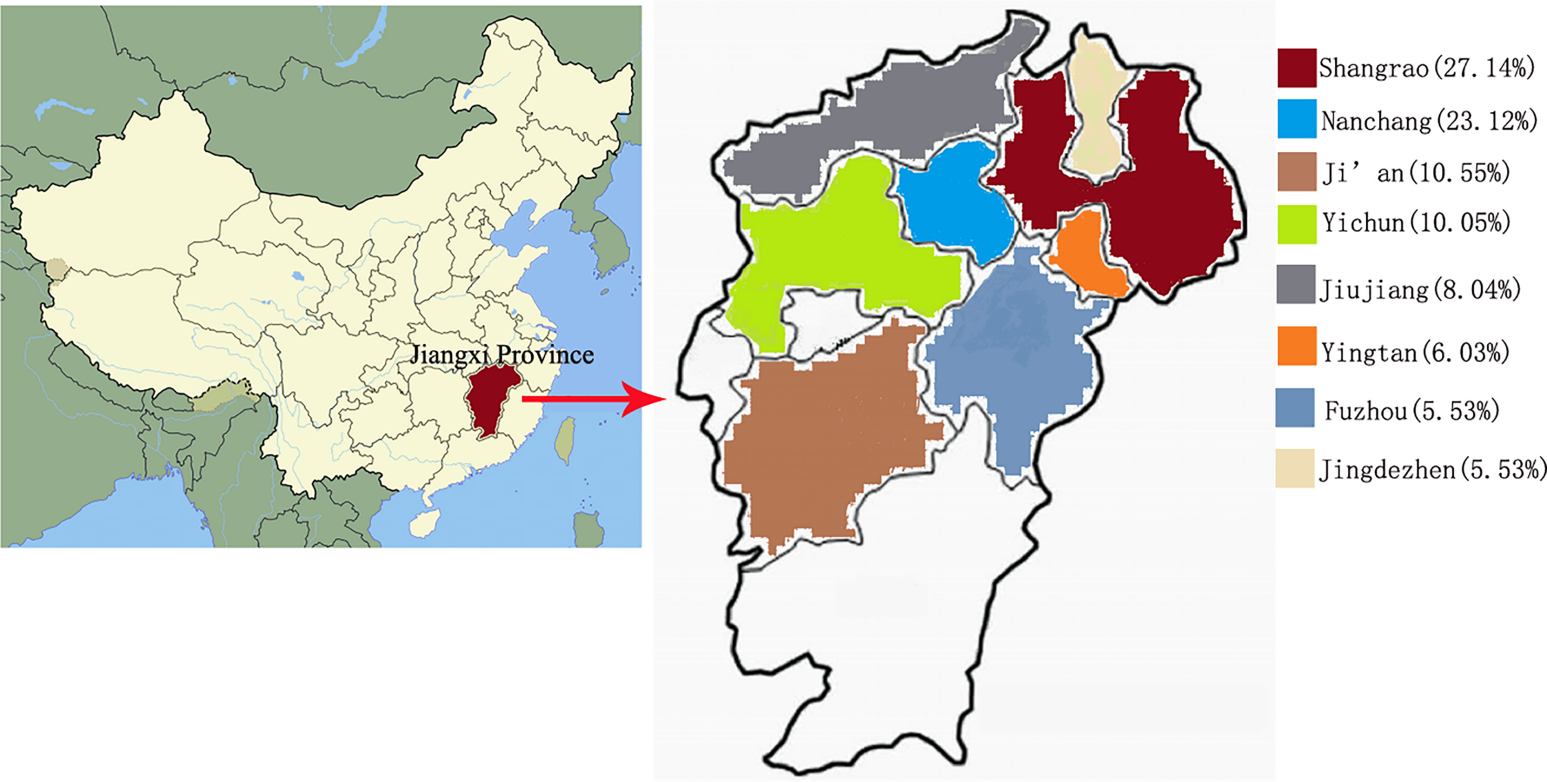
Surveillance point, Jiangxi Province and patient distribution in various cities.

The investigated area, Jiangxi, is located in South Central China on the south bank of the middle and lower reaches of the Yangtze River (**Figure 1**). The location is characterized by an annual 77.5% humidity and 18.75°C temperature, which corresponds to a typical subtropical climate. The data were checked by two highly qualified physicians (ZP and CM), including the epidemiological, demographic, clinical, and laboratory examinations. The inclusion criteria were as follows: (1) discharge diagnosis referring to “cryptococcosis”; (2) clinical and radiographic findings consistent with cryptococcosis; (3) positive result of a cryptococcal capsular polysaccharide antigen (CrAg) test; and (4) positive fungal culture and persistence in the microbiology tests (see below).

### Strains and Polymerase Chain Reaction Procedure

All clinical strains were stored in 20% glycerol at -80°C and recovered by culture at 35°C in a Sabouraud dextrose agar (SDA; 1% peptone, 4% glucose, and 1.5% agar) medium containing chloramphenicol (100 µg/ml). Genomic DNA was extracted from each isolate according to the instructions of the manufacturer (plant genomic DNA extraction reagents from KangWei Century Co., product no. CW0531M) with minor modifications. Briefly, the strains were incubated on SDA agar plates at 35°C for 24 hours. Half gram of fungal cells was picked and transferred to a 2-ml Eppendorf tube containing 400 µl of LP1 buffer; the tube was shaken three times by a tissue lapping machine. Then, the mixture was removed, and 5 µl of RNase A was added; subsequent steps followed the instructions of the manufacturer. Finally, the DNA was dissolved in 50 µl of sterilized water and stored at -80°C. PCR was performed with the 2×T5 Super PCR Mix (Qingke Biotechnology Company, TSE005) according to the instructions of the manufacturer.

### Identification of the Species, Serotypes, and Mating Types

To identify the *Cryptococcus* species, we amplified the IGS1 gene regions of all samples. The PCR products were detected after electrophoresis through 1% agarose gels, and positive products were sequenced by Qingke Biotechnology Company on the ABI 3730xL platform. The sequences were edited by the Seqman software and BLAST’ed in the NCBI database. To determine the serotypes and mating types, we used a previously reported method (Yan et al., 2002). Briefly, serotype- and mating type-specific primers (**Table S1**) of the STE20 gene were amplified and checked on 1% agarose gels together with the samples of the reference strains.

### Multiolocus Sequence Typing and Phylogeny Analysis

The present study used seven universal housekeeping genes for the MLST analysis of the *C. neoformans/C. gattii* complex according to the ISHAM consensus criteria (CAP59, GPD1, LAC1, PLB1, SOD1, URA5, and IGS1 regions) (see **Table S1**) (Hiremath et al., 2008; Meyer et al., 2009). Briefly, all genes were amplified according to (Hiremath et al., 2008), sequenced by Qingke Biotechnology Company, and then BLAST’ed in NCBI for confirmation. Then, all sequences were submitted to the NCBI GenBank to acquire the gene accessions and to the *C. neoformans/C. gattii* species complex database (https://mlst.mycologylab.org/page/Home) to obtain the sequence types (ST).

For each ST genotype, we used a single clinical strain for the phylogenetic analysis. The STs reported by Chen et al. (2018) and different from the STs in the present study were downloaded and incorporated into the tree. Each gene was checked by the Mega 6.06 software, and the seven MLST loci were concatenated by Gedit. The phylogenetic tree was constructed by the neighbor-joining algorithm according to the Tamura-Nei model using 500 bootstrap replications with JEC21 as the outgroup (Tamura et al., 2013).

### *In Vitro* Antifungal Susceptibility Testing

According to the antifungal drug sensitivity test protocol CLSI-M27-A3, fluconazole (FLU) drug sensitivity test was performed in a total of 199 C*. neoformans* isolates, and 86 strains with FLU MIC_50_ ≥ 8 μg/ml were tested for antifungal susceptibility to amphotericin B (AMB), flucytosine (5FC), voriconazole (VOR), itraconazole (ITR), and posaconazole (POS). *Candida krusei* ATCC6258 and *Candida parapsilosis* ATCC22019 were used as quality controls. The minimal inhibitory concentrations (MICs) and epidemiological cutoff values (ECVs) were obtained according to the standard manual procedures. The ECV breakpoints for fluconazole and flucytosine were ≥8 μg/ml, as suggested by previous studies (Espinel-Ingroff et al., 2012a; Espinel-Ingroff et al., 2012b), and breakpoints for amphotericin B were ≥1 μg/ml (Espinel-Ingroff et al., 2012b). For ITR, VOR, and POS, we used the value ≥0.25 μg/ml from a previous study (Espinel-Ingroff et al., 2012a).

## Results

### Demographic Data Relevant to Clinical Isolates

The present study recruited 230 cryptococcosis cases based on the clinical records in the last 5 years in Jiangxi Province, South China. A total of 5.2% (12/230) of the patients had a coinfection with *Talaromyces marneffei*. Therefore, 218 samples were used for the subsequent molecular and epidemiological analysis. Amplifications based on all seven MLST primer sets identified the genotypes in 199 samples (see below). Most of the isolates were obtained from cerebrospinal fluid (n = 141; 70.9%), followed by blood (n = 56; 28.1%), and single isolate was obtained from the bone marrow and pleural fluid (n = 1; 0.5%). The isolates were obtained from patients in 11 cities in Jiangxi Province (**Table 1** and **Figure 1**). A total of 54 (27.1%) samples were from Shangrao City, 46 (23.1%) samples from Nanchang City, 21 (10.6%) samples from Ji’an, 20 (10.1%) samples from Yichun, 16 (8%) samples from Jiujiang, 12 (6%) samples from Yingtan, 11 (5.5%) samples from Fuzhou, and 11 (5.5%) samples from Jingdezhen. The remaining 8 (4%) samples were obtained from smaller cities or villages. More than 50% of the patients were from the two main cities, Shangrao and Nanchang; Nanchang harbors the central surveillance station for fungal infections in Jiangxi Province. Fewer cases were from Ganzhou, which is 400 km away from Nanchang and has its own large general hospital.

**Table 1:** Clinical information of 199 cryptococcus neoformans infections.

Location	Shangrao		54 (27.1%)
	Nanchang		46 (23.1%)
	Ji’an		21 (10.6%)
	Yichun		20 (10.1%)
	Jiujiang		16 (8%)
	Yingtan		12 (6%)
	Fuzhou		11 (5.5%)
	Jingdezhen		11 (5.5%)
	Xinyu		4 (2%)
	Ganzhou		3 (1.5%)
	Pingxiang		1 (0.5%)
Gender	Male		128 (64.3%)
	Female		71 (35.7%)
Specimen	Cerebrospinal fluid (CSF)		141 (70.9%)
	Blood		56 (28.1%)
	Hydrothorax		1 (0.5%)
	Bone marrow		1 (0.5%)
Immune status	Immunocompromised 59.30% (118/199)	HIV	61.9% (73/118)
		Other opportunistic infections	40.7% (48/118)
		Diabetes	22% (26/118)
		Autoimmune systemic diseases	7.6% (9/118)
		Others	9.3% (11/118)
	Immunocompetent	40.7% (81/199)	

A total of 199 isolates included 128 (64.3%) isolates from male patients and 71 (35.7%) isolates from female patients. The age of the patients ranged from 4 to 79 years, with a mean age of 48.8 ± 17 (M ± SD) years. The following age distribution was detected: 3 patients younger than 18 years, 79 aged 19–45 years, 94 aged 46–70 years, and 23 older than 70 years.

In total, 59.3% (118/199) of the patients had a deficient or suppressed immune status, and 40.7% (81/199) were apparently immunocompetent. A total of 36.7% (73/199) of the patients had an HIV-related pathology, 13.1% (26/199) had diabetes, 24.1% (48/199) had other opportunistic infections, i.e., cytomegalovirus infection and *Mycobacterium tuberculosis*, 4.5% (9/199) had a systemic autoimmune disease, and the remaining 5.5% (11/199) were immunosuppressed with other diseases, including malignant cancer and leukopenia. All patients received timely antifungal treatment in combination with supportive therapy after the diagnosis of cryptococcosis; however, 8 patients died due to severe organ damage.

### Identification and Analysis of the Serotype, Mating Type, and Multilocus Sequence Typing

Initially, genomic DNA of the strains was extracted, and the IGS1 gene was amplified. BLASTn search against the NCBI database identified 218 isolates as *C. neoformans* species. A total of 199 strains were identified at the level of the genotypes by MLST analysis. The 12 isolates, coinfected with *Talaromyces marneffei*, were not further explored*. C. gattii* strains were not detected. The serotype and mating type of all these isolates were determined by the methods described by Yan et al. (2002). The primer sequences are listed in **Table S1**. A 588-bp fragment of all 199 strains was amplified by using the STE20Aα primer pair. All tests with the STE20Aa, STE20Da, and STE20Dα primer pairs were negative. All strains were determined to be serotype A and mating type α.

MLST analysis identified eight different sequence types (STs). A total of 178 (89.5%) isolates belonged to ST5, 9 to ST359, 4 to ST6, 1 to ST31, and 1 to ST81. Three novel sequence types were detected in six isolates with previously unknown sequence variations. The new sequence variations matched the ST656, ST657, and ST658 designations from the Fungal MLST Database (https://mlst.mycologylab.org).

Allelic assignments of the gene sequences in the MLST database for eight multilocus sequence types are listed in **Table 2**. The ST5 genotype was predominant and widely distributed in Jiangxi Province. Nine ST359 strains originated from Ji’an (3), Shangrao (2), Nanchang (2), Jiujiang (1), and Xinyu (1). All four ST6 strains originated from Shangrao; a single ST81 strain originated from Yichun, and a single ST31 strain originated from Ganzhou. Three ST657 strains with novel genotypes were from Shangrao; two ST658 strains from Yichun, and the ST656 strain from Nanchang.

**Table 2:** Allelic assignments of eight multilocus sequence types in the present study.

Sequence Type	CAP59	GPD1	IGS1	LAC1	PLB1	SOD1	URA5	Isolates amount
ST5	1	3	1	5	2	1	1	178
ST6	1	1	1	3	2	1	5	4
ST31	1	1	10	3	2	1	1	1
ST81	1	1	1	5	2	1	1	1
ST359	1	25	1	5	2	1	1	9
ST656	1	25	1	5	2	68	1	1
ST657	1	25	1	5	44	1	1	3
ST658	1	3	98	5	2	1	1	2

### Phylogenetic Analysis

**Figure 2:**
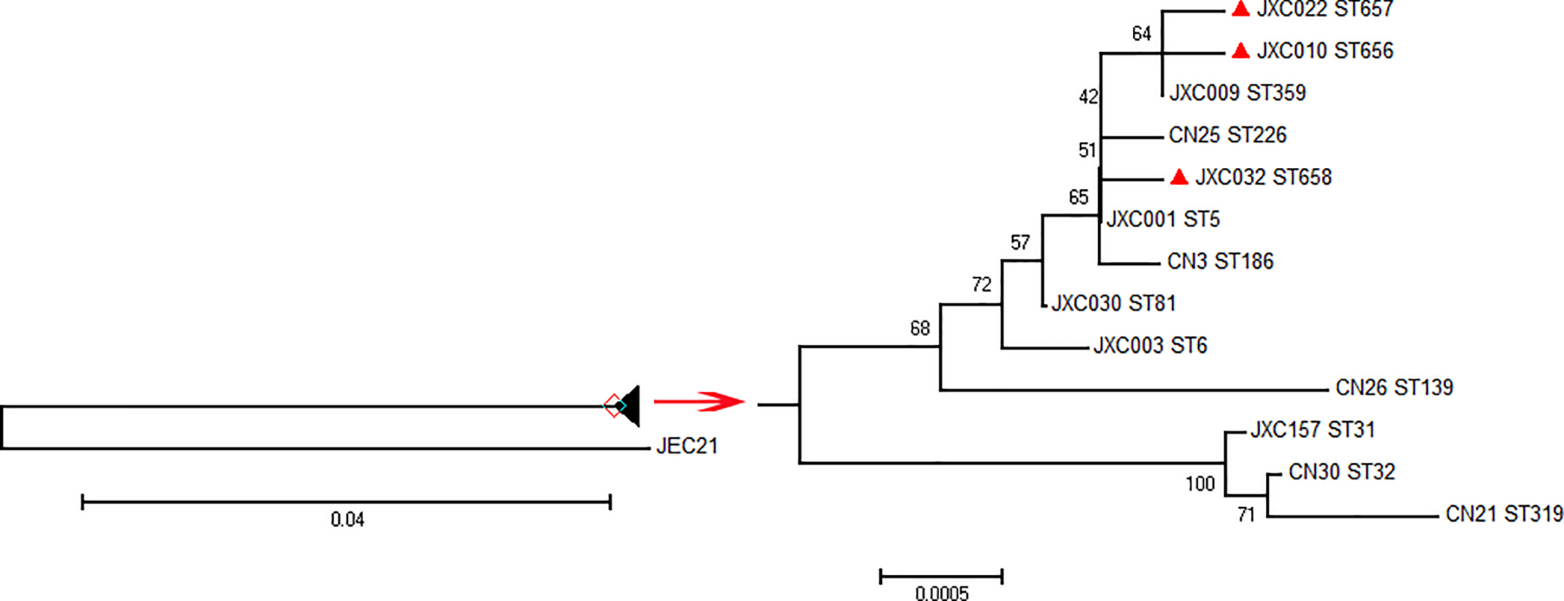
Phylogenetic tree constructed using the neighbor-joining method with a bootstrap of 500 based on the concatenated sequences at seven MLST loci using JEC21 as the outgroup.

A combination with the data reported in a study of Chen et al. (2018) indicated that 13 ST types were present in Jiangxi Province, including ST5, ST6, ST31, ST32, ST81, ST139, ST186, ST226, ST319, ST359, ST656, ST657, and ST658. The results of the present study indicated that ST31, ST32, and ST319 were genetically similar and clustered into a clade separate from the other strains. The other strains were more similar to each other, having only a few nucleotide polymorphisms (**Figure 2**).

### Details of a New Sequence Type Genotype

The present study detected three novel genotypes (ST656, ST657, and ST658), which were different from the previously known STs in the loci SOD1, PLB1, and IGS1. Detailed allelic assignment of each new sequence type was as follows: SOD1: allele #68 was detected in ST656; PLB1: allele #44 in ST657; and IGS1: allele #98 in ST658. The three new genotypes were scattered in various regions, and the patients had different underlying diseases. The ST656 strain originated from the CSF of a 79-year-old male patient who lived in Nanchang and had concomitant complications, including pulmonary tuberculosis, hypertension, gout disease, and chronic hepatitis B infection. Five other patients were immunocompromised and had HIV infection, diabetes, cancer, or surgery. However, none of these patients reported a history of travel or close contact with pigeons.

### *In Vitro* Antifungal Susceptibility

The drug sensitivity test to fluconazole (FLU) was performed using 199 C*. neoformans* isolates, including 113 (56.8%) isolates that had an MIC_50_ < 8 μg/ml, 64 (32.2%) that had an MIC_50_ of 8 μg/ml, and 22 (11.1%) that had an MIC_50_ ≥ 16 μg/ml (**Table 3**). Recommended breakpoints for *Cryptococcus* sensitivity to FLU are undefined; thus, we regarded 86 strains (43.2%) with an MIC_50_ 0 ≥8 μg/ml as resistant strains and tested their susceptibility to other commonly used antifungal drugs, including amphotericin B (AMB), flucytosine (5-FC), voriconazole (VOR), itraconazole (ITR), and posaconazole (POS).

**Table 3:** The results of the *in vitro* tests of six antifungal drugs in 86 fluconazole-resistant isolates.

Drugs	Break points (μg/ml)	Isolates amount		Genotype
FLU	≥16	22	–	ST5 (15), ST6 (3), ST359 (2)
	8	–	64	ST5(56), ST81(1), ST359 (4), ST656(1), ST657(1), ST658 (1)
ITR	≥0.25	85		ST5(73), ST6(3), ST81(1), ST359(5), ST656(1), ST657(1), ST658 (1)
AMB	≥1	69		ST5(59), ST6(2), ST81(1), ST359(5), ST657(1), ST658 (1)
POS	≥0.25	83		ST5(72), ST6(3), ST81(1), ST359(5), ST657(1), ST658(1)
VOR	≥0.25	6		ST5(4), ST6(2)
5FC	≥8	18		ST5(14), ST6(1), ST359(2), ST658(1)

MIC_50_ for FLU ranged from 0.25 to 64 μg/ml (GM 5.1 μg/ml). The percentages of resistant strains in each location were as follows: 40.7% (22/54) in Shangrao, 45.7% (21/46) in Nanchang, 38.1% (8/21) in Ji’an, 55% (11/20) in Yichun, 50% (8/16) in Jiujiang, 50% (6/12) in Yingtan, 54.6% (6/11) in Jingdezhen, 66.7% (2/3) in Ganzhou, 9.1% (1/11) in Fuzhou, and 25% (1/4) in Xinyu. These FLU-resistant strains had a highly variable ST genotype distribution: 39.9% (71/178) of ST5, 75% (3/4) of ST6, 66.7% (6/9) of ST359, 33% (1/3) of ST657, 50% (1/2) of ST658, and 100% of ST81 (1/1) and ST656 (1/1). Considering the high variability in the ST strain percentages, it is difficult to define the relationship between FLU resistance and the ST genotypes.

Our data indicated that nearly all 86 fluconazole-resistant *Cryptococcus* isolates were also resistant to ITR and POS, most of the 86 fluconazole-resistant *Cryptococcus* isolates were resistant to AMB. Only three strains (JXC070, JXC010, and JXC136) were susceptible to POS; a single strain (JXC136) was susceptible to ITR (**Table 3**).

These 86 strains included 18 strains that were not susceptible to 5-FC (JXC008, JXC013, JXC016, JXC017, JXC019, JXC032, JXC038, JXC054, JXC070, JXC081, JXC096, JXC113, JXC145, JXC146, JXC181, JXC254, JXC260, and JXC278), and 6 strains were not sensitive to VOR (JXC001, JXC003, JXC038, JXC054, JXC143, and JXC191). A total of 22 strains with an FLU MIC_50_ ≥ 16 μg/ml included 7 isolates (JXC017, JXC019, JXC038, JXC054, JXC081, JXC113, and JXC145) resistant to 5-FC and 5 isolates (JXC003, JXC038, JXC054, JXC143, and JXC191) to VOR (**Table 3**).

## Discussion

An increasing number of studies on the epidemiology of cryptococcosis in China have been published; however, only a very few reports described the situation in Jiangxi Province, which is a large agricultural province with a population of more than 45 million people in South Central China (Chen et al., 2008). Only a single molecular epidemiology study on cryptococcosis was reported in 2018, in which Chen et al. (2018) analyzed 86 clinical cryptococcosis cases in this region. *C. gattii* was not detected, and all strains were sensitive to flucytosine, amphotericin B, fluconazole, itraconazole, and voriconazole (Chen et al., 2018). The study has been the first to report the profiles of *Cryptococcus* infections in the Jiangxi region; however, some questions remained and needed to be confirmed using large-scale data.

The present study recruited 230 cases of cryptococcosis over 5 years at two hospitals. One of the hospitals was a large general hospital (Jiangxi Provincial People’s Hospital with more than 3,500 ward beds), and another hospital was a specialized hospital (Jiangxi Provincial Chest Hospital), which is famous for treating cryptococcosis and registered the highest number of *Cryptococcus* cases in Jiangxi Province. To a certain extent, these data can reveal the general distribution of this disease.

*C. neoformans* s.l. is globally distributed, and *Cryptococcus gattii* s.l. has a wide distribution with hot spots in America, Australia, and Sub-Saharan Africa. Recently, Cao et al. reported an emergence of *C. gattii* s.l. infections in Guangxi in South China (Huang et al., 2020) However, the present study and the study of Chen et al. (2018) did not detect the strains of *C. gattii* s.l. in Jiangxi. All 199 strains belonged to the serotype A and MATα. Monitoring should be continued to prevent the invasion and spreading of *C. gattii* s.l.

Usually, cryptococcosis is an opportunistic fungal disease that mainly infects immunocompromised populations, particularly HIV-infected patients. However, the situation seems to be rather different in China. A review by Chen et al. published in 2020 pointed out that the proportion of HIV-related cryptococcosis meningitis (CM) is 80% in the United States, 95% in Brazil, 77% in Europe, and only 16% in China (Viviani et al., 2006; Leal et al., 2008; Pyrgos et al., 2013; Chen et al., 2020). The authors speculated that the reported differences are likely due to a biased reporting in China, and most cases have been reported by university-affiliated hospitals, which are not officially designated specialized hospitals that treat highly infectious diseases, i.e., HIV and tuberculosis. Therefore, the authors suggested that occurrence of CM in HIV-infected population in China has been severely underreported (Chen et al., 2020). The results of the present study indicated that HIV-positive patients accounted for 36.7% of all cases, and approximately 60% of the patients had an underlying deficiency or suppressed immune system; these numbers were considerably higher than the numbers reported previously. Considering that most of the cases of the present study originated from Jiangxi Provincial Chest Hospital, which is famous for treating cryptococcosis and, therefore, attracts the highest number of cases of this disease, the results of the present study confirmed the suggestion of Chen on the enormous underreporting of HIV-related cryptococcosis infections.

To date, a total of 17 ST genotypes have been reported in China, including 15 sequence types (ST5, ST31, ST38, ST53, ST57, ST63, ST93, ST186, ST191, ST194, ST195, ST295, ST296, ST359, and ST360) in mainland China and another two types (ST4 and ST6) from Hong Kong, China (Khayhan et al., 2013; Dou et al., 2015; Fan et al., 2016; Dou et al., 2017). Here, we reported three new STs (ST356, ST357, ST358), but according to the phylogenetic analysis, they are very similar to the existing genotypes and only one nucleotide differentiated in each new ST. Cryptococcosis in China is mainly caused by *C. neoformans*, and >90% of the strains belong to ST5 (Fan et al., 2016). Combined analysis of the sequences reported by Chen et al. (2018) indicated that 13 STs were identified in Jiangxi by a consensus multilocus sequence typing protocol, and three novel ST genotypes (ST656, ST657, and ST658) were detected in the present study and acquired from the Fungal MLST Database (https://mlst.mycologylab.org). Similar to a report of Chen et al. (2018), 90% of the strains in the present study belonged to ST5 (molecular type VNI), serotype A, and mating type α (MAT α). Therefore, these data indicated that serotype A and MAT α strains of the ST5 genotype are predominant in Jiangxi Province. *C. neoformans* is very successful at long-distance and short-distance dispersal, and abundant genetic mutant strains may be present in Jiangxi and may have unique genes. The spread of the genotypes between various geographical locations may be caused by wind, pigeons and other animals, plants, and human activities. Genetic mutations may be a reason for the presence of new sequence types in Jiangxi.

Mainly three classes of agents are used to treat *Cryptococcus* infections: polyenes (mainly amphotericin B), azoles (mainly fluconazole), and nucleoside analog (mainly 5-flucytosine). Amphotericin B preparations plus 5-flucytosine is often used as the initial treatment of meningitis, disseminated infection, or moderate-to-severe pulmonary infection followed by fluconazole as a consolidation therapy. Resistance against these substances have been detected in several *Cryptococcus* strains. The general molecular mechanisms of resistance are conserved among fungal species but have been acquired independently in several fungal strains and taxa. The most prevalent mechanism of resistance involves increased drug efflux pump activity and alterations in antifungal drug targets, due to increased target expression or mutations within the target protein sequence (Berman and Krysan, 2020).

Fluconazole is the most commonly used antifungal agent for the treatment and prophylaxis of cryptococcosis. Over the years, resistance of the clinical isolates of *C. neoformans* to FLU has gradually increased, and current resistance is a relatively common event in relapse episodes of cryptococcal meningitis (Bongomin et al., 2018). In contrast to the report of Chen et al. (2018), which demonstrated that all isolates were sensitive to routine antifungal agents, antifungal susceptibility tests in the present study indicated that 43.2% of the strains had MICs ≥ 8 μg/ml for fluconazole and 11.1% of the strains had MICs higher than ≥16 μg/ml. Most of the strains with MICs ≥ 8 μg/ml were also resistant to ITR, AMB, and POSA, and a few of these strains were resistant to VRC and 5FC. Fluconazole resistance has been reported by a CHIF-NET study in 2018 in China, in which fluconazole-resistant isolates of *C. neoformans* were detected in 9.7% of the strains.

We observed variations in MICs between various ST genotype strains. Approximately 40% of ST5 isolates were not sensitive to FLU with MIC_50_ ≥ 8 μg/ml; for ST6, ST81, ST359, ST656, and ST658 this was 75%, 100%, 66.7%, 100%, and 50%, respectively. Considering the limited data on these ST strains, we cannot determine a relationship between these genotypes and antifungal sensitivity.

In conclusion, the present study on 199 clinical cryptococcosis cases identified ST5 (molecular type VNI) belonging to serotype A and mating type α (MAT α) as the predominant *C. neoformans* in Jiangxi Province, South Central China. High prevalence of immunocompromised-related infections, particularly in the HIV-infected populations, was reported in Jiangxi Province. Additionally, an increased resistance of *C. neoformans* species to fluconazole was detected in this region. Therefore, a close and continuous monitoring of the epidemiology of this severe fungal disease is necessary for public surveillance and precise treatment.

## Data Availability Statement

The original contributions presented in the study are included in the article/**Supplementary Material**. Further inquiries can be directed to the corresponding authors.

## Author Contributions

PZ and MC designed the research. CY, ZB, and FD performed the research. CY, ZB, OB, HC, YL, and YY analyzed the data CY, OB, and PZ wrote the paper. All authors contributed to the article and approved the submitted version.

## Funding

This work was supported by the National Natural Science Foundation of China [81960367], Project of Health Commission of Jiangxi Province (202130956), and Key R&D Project of Science and Technology Department of Jiangxi Province (20203BBG73040).

## Conflict of Interest

The authors declare that the research was conducted in the absence of any commercial or financial relationships that could be construed as a potential conflict of interest.

## Publisher’s Note

All claims expressed in this article are solely those of the authors and do not necessarily represent those of their affiliated organizations, or those of the publisher, the editors and the reviewers. Any product that may be evaluated in this article, or claim that may be made by its manufacturer, is not guaranteed or endorsed by the publisher.

## Supplementary Material

Supplementary Table 1 PCR primers used in the present study.

Supplementary Table 2 Detailed information of the 199 clinical isolates of *Cryptococcus neoformans* from Jiangxi Province, China.

Supplementary Table 3 Susceptibilities of the 86 C*. neoformans* isolates from Jiangxi Province against six common antifungal drugs.

Supplementary Table 4 The list of gene bank accession numbers for seven genes of 199 isolates.
