# Supplementary material for: High Prevalence of HIV-Related Cryptococcosis and Increased Resistance to Fluconazole of the Cryptococcus neoformans Complex in Jiangxi Province, South Central China
Source: Front Cell Infect Microbiol. 2021 Nov 1;11:723251. doi: 10.3389/fcimb.2021.723251 (PMC8592285; doi:10.3389/fcimb.2021.723251)
Supplement: Supplementary Table 1 — PCR primers used in the present study. [file DataSheet_1.pdf]

Table S1. PCR primers used in the present study

Primers	Sequence (5'→3')	Annealing temp (℃)	Expected size (bp)
IGS1-F	ATCCTTTGCAGACGACTTGA	55	~950
IGS1-R	GGGACCAGTGCATTGCATGA		
CAP59 -F	TCTCTACGTCGAGCAAGTCAA	55	~550
CAP59-R	TCCGCTGCACAAGTGATACCC		
GPD1-F	CCACTGAACCCTTCTAGGAGT	55	~670
GPD1-R	CTTCTTGGCACCTCCCTTGAG		
LAC1-F	ACGACGAGGGAGTATACCTTCG	55	~850
LAC1-R	CAATTGACTGGCACGTGAATCT		
PLB1-F	CTTCAGGCGGAGAGAGGTTT	55	~630
PLB1-R	GATTTGGCGTTGGTTTCAGT		
SOD1-F	TCTAATCGAAATGGTCAAGG	55	~660
SOD1-R	CGCAGCTGTTCGTCTGGATA		
URA5-F	ATGTCTTCCCAAGCCCTCGAC	55	~800
URA5-R	TTAAGACCTCTGAACACCGTACTC		
STE20Aa-F	TCCACTGGCAACCCTGCGAG	55	865
STE20Aa-R	ATCAGAGACAGAGGAGCAAGAC		
STE20Aα-F	CCAAAAGCTGATGCTGTGGA	55-45	588
STE20Aα-R	AGGACATCTATAGCAGAT		
STE20Da-F	GATCTGTCTCAGCAGCCAC	60	440
STE20Da-R	AATATCAGCTGCCCAGGTGA		
STE20Dα-F	GATTTATCTCAGCAGCCACG	61	443
STE20Dα-R	AAATCGGCTACGGCACGTC		
