# Supplementary material for: High Prevalence of HIV-Related Cryptococcosis and Increased Resistance to Fluconazole of the Cryptococcus neoformans Complex in Jiangxi Province, South Central China
Source: Front Cell Infect Microbiol. 2021 Nov 1;11:723251. doi: 10.3389/fcimb.2021.723251 (PMC8592285; doi:10.3389/fcimb.2021.723251)
Supplement: Supplementary Table 2 — Detailed information of the 199 clinical isolates of Cryptococcus neoformans from Jiangxi Province, China. [file DataSheet_2.pdf]

Table S2. Detailed information of the 199 clinical isolates of *Cryptococcus neoformans* from Jiangxi Province, China

Isolate	Location	Sex	Age	Specimen	Underlying conditions	ST[a]	Serotype	MT[b]
JXC001	Yingtan	Female	49	CSF	HIV(+)	5	A	α
JXC002	Ji'an	Male	22	CSF	HIV(+)	5	A	α
JXC003	Shangrao	Male	29	CSF	HIV(+)	6	A	α
JXC004	Ganzhou	Female	50	CSF	HIV(+)	5	A	α
JXC005	Fuzhou	Female	46	Blood	Bacterial pneumonia; Systemic lupus erythematosus; Lupus nephritis; Hyperlipemia; Diabetes(typeII)	5	A	α
JXC006	Shangrao	Male	62	CSF	Unknown	5	A	α
JXC008	Nanchang	Female	28	Blood	HIV(+); Bacterial pneumonia; Leukopenia; Hypokalemia	5	A	α
JXC009	Xinyu	Female	27	CSF	HIV(+); Pulmonary tuberculosis	359	A	α
JXC010	Nanchang	Male	79	CSF	Pulmonary tuberculosis; Hypertension; Arthrolithiasis; Hepatitis B	656	A	α
JXC011	Jiujiang	Male	28	CSF	Unknown	5	A	α
JXC012	Ji'an	Male	67	CSF	Pulmonary tuberculosis;	5	A	α
JXC013	Ji'an	Female	51	Blood	Systemic lupus erythematosus; Hypertension	359	A	α
JXC014	Yichun	Male	44	Blood	HIV(+)	5	A	α
JXC016	Ji'an	Female	51	CSF	Systemic lupus erythematosus; Hypertension	359	A	α
JXC017	Yingtan	Male	40	CSF	Hepatitis B; Tuberculosis	5	A	α
JXC018	Shangrao	Male	55	CSF	HIV(+)	5	A	α
JXC019	Jiujiang	Male	49	CSF	Renal dysfunction	5	A	α
JXC020	Jingdezhen	Male	23	Blood	HIV(+);	5	A	α
JXC021	Fuzhou	Male	33	CSF	HIV(+); Bacterial pneumonia; Cytomegalovirus infection; Perianal abscess; Hypohepatia; Drug induced myelosuppression; Hypokalemia	5	A	α

JXC022	Shangrao	Female	63	CSF	HIV(+); Cervical cancer with panhysterectom	657	A	α
JXC023	Ji'an	Female	62	CSF	Unknown	5	A	α
JXC025	Ji'an	Female	28	Blood	Kidney failure	5	A	α
JXC026	Jingdezhen	Male	23	Blood	HIV(+); Cytomegalovirus infection	5	A	α
JXC027	Jingdezhen	Male	23	Blood	HIV(+)	5	A	α
JXC028	Shangrao	Male	49	CSF	HIV(+)	5	A	α
JXC030	Yichun	Male	67	CSF	Bacterial pneumonia; Thrombocytopenia	81	A	α
JXC031	Yichun	Male	67	CSF	Bacterial pneumonia; Thrombocytopenia	5	A	α
JXC032	Yichun	Male	50	CSF	Unknown	658	A	α
JXC033	Fuzhou	Female	63	CSF	Arthritis; Tuberculosis	5	A	α
JXC034	Ji'an	Female	51	CSF	Systemic lupus erythematosus	359	A	α
JXC035	Jingdezhen	Male	61	Blood	HIV(+)	5	A	α
JXC036	Xinyu	Female	27	CSF	Unknown	5	A	α
JXC037	Jingdezhen	Male	61	Blood	HIV(+)	5	A	α
JXC038	Shangrao	Male	29	Blood	Unknown	6	A	α
JXC039	Nanchang	Male	42	CSF	Cerebral infarction; Bacterial pneumonia; Respiratory failure; Diabetes(typeII); Sarcoidosis; Nasopharyngeal cancer	5	A	α
JXC040	Jingdezhen	Male	61	Blood	HIV(+)	5	A	α
JXC041	Yingtan	Male	51	CSF	Cerebral infarction; Pneumocystis	5	A	α
JXC042	Nanchang	Male	42	CSF	Acute myocardial infarction; Cerebral infarction; Bacterial pneumonia; Diabetes(typeII); Nasopharynx cancer; Abnormal liver function	5	A	α
JXC043	Nanchang	Female	74	Blood	Surgery	5	A	α
JXC044	Nanchang	Female	74	CSF	Hip replacement	5	A	α

JXC045	Ganzhou	Female	50	CSF	Unknown	5	A	α
JXC046	Yichun	Female	43	CSF	Drug hepatitis	5	A	α
JXC047	Shangrao	Male	29	Blood	Unknown	6	A	α
JXC048	Nanchang	Female	34	Blood	Respiratory failure; Bacterial pneumonia; Multiple organ dysfunction syndrome	359	A	α
JXC049	Nanchang	Male	59	Blood	HIV(+); Bacterial pneumonia; OralCandidiasis	5	A	α
JXC050	Nanchang	Male	42	CSF	Diabetes(typeII); Sarcoidosis; Nasopharynx cancer	5	A	α
JXC051	Jingdezhen	Male	61	CSF	HIV(+)	5	A	α
JXC052	Fuzhou	Female	51	Blood	HIV(+); Bacterial pneumonia; Renal dysfunction; Anemia; Hepatic cyst; Nephrocystosis	5	A	α
JXC054	Shangrao	Male	47	Blood	Cerebral infarction; Bacterial pneumonia; pulmonary tuberculosis; Hepatitis B	5	A	α
JXC055	Ji'an	Male	54	CSF	Myelodysplastic syndrome with glucocorticoid therapy; Diabetes(typeII)	5	A	α
JXC056	Jiujiang	Male	41	Blood	HIV(+); Cytomegalovirus infection	5	A	α
JXC057	Nanchang	Male	58	CSF	Cerebral ischemia; Hypertension; Diabetes(typeII)	5	A	α
JXC058	Jingdezhen	Male	61	Blood	HIV(+)	5	A	α
JXC059	Yichun	Male	36	CSF	HIV(+); Cytomegalovirus infection	5	A	α
JXC060	Nanchang	Female	25	CSF	Systemic lupus erythematosus; Hypothyroidism	5	A	α
JXC061	Nanchang	Male	44	Blood	HIV(+); Tuberculosis of intestines; Hepatitis B; Pulmonary tuberculosis	5	A	α
JXC062	Jiujiang	Male	48	CSF	Pneumocystis	5	A	α
JXC065	Yichun	Male	36	Blood	HIV(+); Pneumocystis; Cytomegalovirus infection	5	A	α
JXC066	Jiujiang	Female	67	CSF	Hypertension; Diabete2(typeII); Stomach cancer; Diarrhea; Electrolyte disturbance; Hypoproteinemia; Fever	5	A	α
JXC068	Yingtan	Female	79	CSF	Diarrhea; Hypertension	5	A	α

JXC070	Ji'an	Male	25	CSF	HIV(+); Cerebral hernia; Bacteremia; Suppurative meningitis; Bacterial pneumonia; Electrolyte disturbance; Hyponatremia; Leukopenia; Thrombocytopenia; Anemia	5	A	α
JXC071	Nanchang	Male	44	Blood	HIV(+); Tuberculosis of intestines; Hepatitis B; Pulmonary tuberculosis	5	A	α
JXC075	Shangrao	Male	23	Blood	Cerebral hernia	5	A	α
JXC078	Yichun	Male	44	CSF	Hyponatremia; Hepatitis B	5	A	α
JXC081	Yichun	Female	32	CSF	Unknown	5	A	α
JXC084	Jiujiang	Male	52	Blood	HIV(+); Pneumocystis; Cytomegalovirus infection; Pneumonia; Oral Candidiasis; Bacterial pneumonia; Respiratory failure; Hypoproteinemia	5	A	α
JXC087	Xinyu	Male	76	CSF	Malignant lymphoma; Bacterial pneumonia; Renal dysfunction; Hypoproteinemia; Hypokalemia; Hypohepatia	5	A	α
JXC088	Yichun	Male	45	CSF	HIV(+); Bacterial pneumonia; Cerebral hernia; Epilepsy; Hypohepatia; Hypokalemia	5	A	α
JXC091	Shangrao	Male	23	CSF	Cerebral hernia	5	A	α
JXC092	Yichun	Male	45	Blood	HIV(+); Cerebral hernia; Epilepsy; Hypohepatia; Hypokalemia	5	A	α
JXC095	Shangrao	Male	23	Blood	Bacterial pneumonia	5	A	α
JXC096	Shangrao	Male	23	Blood	Oral Candidiasis	5	A	α
JXC097	Jiujiang	Male	52	Blood	HIV(+); Pneumo cystis pneumonia; Cytomegalovirus infection; Oral Candidiasis; Bacterial pneumonia; Respiratory failure; Hypoproteinemia	5	A	α
JXC098	Shangrao	Male	23	Blood	Cerebral hernia	5	A	α
JXC101	Ji'an	Male	50	Blood	Engaged in animal husbandry;	5	A	α
JXC102	Yichun	Male	36	Blood	HIV(+); Pneumocystis; Cytomegalovirus infection	5	A	α
JXC104	Shangrao	Female	47	CSF	Bacterial pneumonia; Kidney stones	359	A	α
JXC105	Yingtan	Male	69	CSF	Respiratory failure; Shock; Multiple organ dysfunction syndrome;	5	A	α

					Electrolyte disturbance; Lactic acidosis; Hypoproteinemia			
JXC107	Ji'an	Male	65	CSF	Hypertension; Bronchiectasis with infection	5	A	α
JXC109	Shangrao	Female	67	CSF	Arrhythmia; Abnormal liver function	5	A	α
JXC110	Ji'an	Male	50	CSF	Unknown	5	A	α
JXC111	Yichun	Female	61	CSF	Bacterial pneumonia	5	A	α
JXC113	Nanchang	Male	73	Blood	Cerebral infarction; Bacterial pneumonia; Hypoproteinemia; Electrolyte disturbance	5	A	α
JXC114	Shangrao	Female	65	CSF	Pneumocystis; Diabetes(typeII)	5	A	α
JXC115	Nanchang	Female	25	CSF	Primary hypothyroidism; Systemic lupus erythematosus	5	A	α
JXC123	Nanchang	Male	17	CSF	Insomnia	5	A	α
JXC124	Yichun	Female	61	CSF	Unknown	5	A	α
JXC125	Nanchang	Female	25	CSF	Systemic lupus erythematosus; Idiopathichy pothyroidism	5	A	α
JXC126	Nanchang	Female	73	CSF	Bacterial pneumonia; Cerebral infarction; Tuberculosis; Hypertension	5	A	α
JXC129	Nanchang	Female	73	CSF	Bacterial pneumonia; Tuberculosis; Hypertension	5	A	α
JXC130	Shangrao	Male	71	Blood	Bacterial meningitis; Spontaneous pneumothorax; Renal dysfunction; Hypoproteinemia; Lower gastrointestinal bleeding; Electrolyte disturbance; Hypertension; Diabetes(typeII)	5	A	α
JXC132	Nanchang	Female	73	Blood	Bacterial pneumonia; Cerebral infarction; Tuberculosis; Hypertension	5	A	α
JXC133	Nanchang	Female	36	Blood	HIV(+)	5	A	α
JXC134	Nanchang	Female	28	Blood	HIV(+); Leukopenia; Hypokalemia	5	A	α
JXC135	Nanchang	Female	36	CSF	HIV(+); Cerebral hernia; Shock	5	A	α
JXC136	Shangrao	Male	66	Blood	HIV(+); Disseminated Talaromycosis; Bacterial pneumonia; Cytomegalovirus infection; Thrombocytopenia; Anemia; Leukopenia;	359	A	α

					Electrolyte disturbance; Hypoproteinemia			
JXC138	Shangrao	Male	65	CSF	Pneumocsticosis	5	A	α
JXC140	Fuzhou	Male	33	CSF	HIV(+); Bacterial pneumonia; Cytomegalovirus infection; Perianal abscess; Hypohepatia; Drug induced myelosuppression; Hypokalemia	5	A	α
JXC141	Shangrao	Female	75	CSF	Electrolyte disturbance; Cerebral ischemia	5	A	α
JXC143	Shangrao	Male	71	CSF	Bacterial meningitis; Renal dysfunction; hypoproteinemia; Electrolyte disturbance; Hypertension; Diabetes(typeII)	5	A	α
JXC144	Nanchang	Male	73	Blood	Cerebral infarction; Bacterial pneumonia; Hypoproteinemia; electrolyte disturbance	5	A	α
JXC145	Shangrao	Male	71	CSF	Spontaneous pneumothorax; Renal dysfunction; Hypoproteinemia; Lower gastrointestinal bleeding; Electrolyte disturbance; Hypertension; Diabetes(typeII)	5	A	α
JXC146	Shangrao	Male	71	Blood	Bacterial meningitis; Renal dysfunction; hypoproteinemia; Lower gastrointestinal bleeding; Electrolyte disturbance; Hypertension; Diabetes(typeII)	5	A	α
JXC147	Yingtan	Male	69	CSF	Bacterial pneumonia; Respiratory failure; Shock; Multiple organ dysfunction syndrome; Electrolyte disturbance; Hypoproteinemia	5	A	α
JXC148	Shangrao	Male	29	CSF	Suppurative meningitis; Cerebral infarction; Hepatitis B; Epilepsy; Hypokalemia	5	A	α
JXC149	Nanchang	Male	55	CSF	Cerebral ischemia; Hyperlipemia	5	A	α
JXC151	Nanchang	Female	74	Blood	Hip replacement	5	A	α
JXC153	Shangrao	Male	26	CSF	HIV(+); Bacterial pneumonia; Leukopenia; Hypoproteinemia	5	A	α
JXC154	Ji'an	Male	22	CSF	HIV(+); Bacterial pneumonia; Cytomegalovirus infection	5	A	α
JXC155	Yichun	Female	43	CSF	Drug hepatitis	5	A	α
JXC157	Ganzhou	Male	64	CSF	Non-Hodgkin's Lymphoma; Bacterial pneumonia	31	A	α

JXC160	Nanchang	Male	59	CSF	Unknown	5	A	α
JXC161	Jiujiang	Male	25	CSF	HIV(+); Drug hepatitis	5	A	α
JXC163	Jiujiang	Male	65	CSF	Rheumatoid Arthritis; Brain Surgery; Pulmonary tuberculosis	5	A	α
JXC164	Shangrao	Male	27	CSF	HIV(+); Bacterial pneumonia; Respiratory failure; Electrolyte disturbance	5	A	α
JXC165	Jiujiang	Female	67	CSF	Connective tissue disease; Diabetes(typeII); Herpes Zoster; Hepatitis B	5	A	α
JXC167	Nanchang	Female	27	CSF	Plastic surgery	5	A	α
JXC169	Yichun	Male	67	CSF	Bacterial pneumonia; Thrombocytopenia	5	A	α
JXC170	Fuzhou	Female	63	CSF	Hepatitis B	5	A	α
JXC171	Shangrao	Male	29	CSF	Bacterial pneumonia	5	A	α
JXC172	Yichun	Male	51	CSF	Epilepsy; Dropsical nephritis; Diabetes(typeII)	658	A	α
JXC173	Fuzhou	Female	63	CSF	Arthritis; Tuberculosis	5	A	α
JXC174	Shangrao	Male	27	Blood	HIV(+); Bacterial pneumonia; Respiratory failure; Electrolyte disturbance	5	A	α
JXC176	Nanchang	Male	26	CSF	HIV(+); Hemiplegia	5	A	α
JXC177	Shangrao	Male	47	CSF	Cerebral infarction; pulmonary tuberculosis	5	A	α
JXC178	Shangrao	Male	47	Blood	Pulmonary tuberculosis; Hepatitis B	5	A	α
JXC179	Nanchang	Female	27	Blood	Facial surgery	5	A	α
JXC180	Shangrao	Male	47	CSF	Cerebral infarction	5	A	α
JXC181	Nanchang	Female	27	CSF	Brain Surgery	5	A	α
JXC183	Xinyu	Male	4	CSF	Unknown	5	A	α
JXC184	Fuzhou	Male	37	CSF	HIV(+)	5	A	α
JXC187	Nanchang	Male	26	CSF	HIV(+); Hemiplegia; Disseminated; Talaromycosis	5	A	α
JXC188	Ji'an	Male	78	CSF	HIV(+); Bacterial pneumonia	5	A	α
JXC189	Nanchang	Female	27	CSF	Anaplasty	5	A	α

JXC191	Yingtan	Male	49	CSF	HIV(+)	5	A	α
JXC192	Nanchang	Female	27	Blood	Trauma	5	A	α
JXC194	Ji'an	Male	78	Blood	HIV(+); Bacterial pneumonia	5	A	α
JXC222	Nanchang	Male	49	CSF	HIV(+); Pneumocystis; Hepatitis B	5	A	α
JXC225	Shangrao	Male	49	CSF	Facial neuritis; Renal dysfunction	5	A	α
JXC226	Fuzhou	Male	33	CSF	Hepatitis B	5	A	α
JXC227	Nanchang	Male	70	CSF	Hypoproteinemia; Hepatitis B	5	A	α
JXC229	Nanchang	Male	76	CSF	Epilepsy	5	A	α
JXC230	Pingxiang	Male	49	CSF	Unknown	5	A	α
JXC231	Yingtan	Male	25	CSF	HIV(+); Herpes zoster	5	A	α
JXC233	Shangrao	Male	26	Blood	HIV(+); Bacterial pneumonia; Leukopenia; Hypoproteinemia	5	A	α
JXC234	Ji'an	Female	36	CSF	Rheumatoid arthritis Glucocorticoid therapy; Rheumatoid arthritis; Diabetes(typeII); Hypokalemia	5	A	α
JXC235	Shangrao	Male	53	CSF	Unknown	5	A	α
JXC236	Shangrao	Female	63	Blood	HIV(+); Trauma	657	A	α
JXC237	Ji'an	Female	48	CSF	HIV(+)	5	A	α
JXC239	Shangrao	Female	56	Blood	HIV(+); Breast cancer with surgery	657	A	α
JXC240	Jiujiang	Female	69	CSF	Unknown	5	A	α
JXC241	Shangrao	Female	55	CSF	HIV(+); Herpes Zoster; Pulmonary tuberculosis; Leukopenia	5	A	α
JXC242	Jingdezhen	Male	42	CSF	Drug hepatitis	5	A	α
JXC243	Nanchang	Male	33	CSF	HIV(+); Pneumocystis; Hepatitis B	5	A	α
JXC244	Nanchang	Male	38	Bone marrow	Arthrolithiasis; Accident fracture; Pulmonary tuberculosis	5	A	α

JXC246	Jingdezhen	Male	38	CSF	Thrombocytopenia	5	A	α
JXC247	Ji'an	Male	78	CSF	HIV(+); Bacterial pneumonia	5	A	α
JXC249	Shangrao	Male	55	CSF	Cerebral infarction; Abnormal liver function; Diabetes(typeII)	5	A	α
JXC250	Ji'an	Female	62	CSF	Bacterial pneumonia	5	A	α
JXC251	Fuzhou	Male	69	CSF	Bacterial pneumonia	5	A	α
JXC252	Fuzhou	Male	69	CSF	Arthrolithiasis	5	A	α
JXC253	Nanchang	Male	17	CSF	Unknown	5	A	α
JXC254	Nanchang	Male	59	CSF	HIV(+); Bacterial pneumonia; Oral Candidiasis	5	A	α
JXC255	Nanchang	Female	78	CSF	Hypertension; Diabetes(typeII); Herpes Zoster; Urinary tract infection		A	α
JXC256	Yingtan	Male	51	CSF	Pneumonia	5	A	α
JXC257	Nanchang	Female	34	Blood	Respiratory failure; Bacterial pneumonia; Multiple organ dysfunction syndrome	359	A	α
JXC258	Shangrao	Male	54	CSF	Cerebral infarction; Abnormal liver function; Diabetes(typeII)	5	A	α
JXC259	Jiujiang	Male	41	CSF	HIV(+)	5	A	α
JXC260	Jiujiang	Male	41	CSF	HIV(+); Cytomegalovirus infection	5	A	α
JXC261	Shangrao	Female	45	CSF	HIV(+); Asthma with Glucocorticoid therapy; Bronchitis	5	A	α
JXC262	Ji'an	Male	42	CSF	HIV(+); Pneumocystis; Cytomegalovirus infection; Drug hepatitis	5	A	α
JXC264	Yichun	Female	42	CSF	HIV(+); Oral and esophageal candidadisis; Bacterial pneumonia; Respiratory failure	5	A	α
JXC265	Yingtan	Male	51	CSF	Pneumonia	5	A	α
JXC266	Yichun	Female	76	CSF	Hypertension; Diabetes(typeII); Urinary tract infection; Hypokalemia	5	A	α
JXC269	Shangrao	Male	29	Blood	HIV(+)	6	A	α
JXC270	Yichun	Female	76	CSF	Hypertension; Diabetes(typeII); Urinary tract infection; Hypokalemia	5	A	α

JXC274	Jiujiang	Female	67	CSF	Hypertension; Diabetes(typeII); Stomach cancer; Diarrhea; Electrolyte disturbance; Hypoproteinemia; Fever	5	A	α
JXC275	Jiujiang	Male	41	CSF	HIV(+); Cytomegalovirus infection	5	A	α
JXC276	Shangrao	Male	59	CSF	Leukopenia; Hypoproteinemia	5	A	α
JXC277	Shangrao	Male	58	CSF	Unknown	5	A	α
JXC278	Jingdezhen	Male	61	CSF	Unknown	5	A	α
JXC279	Shangrao	Female	45	CSF	HIV(+); Asthma; Glucocorticoid therapy; Bronchitis	5	A	α
JXC280	Shangrao	Female	57	CSF	Diabetes(typeII); Hypertension	5	A	α
JXC282	Yingtan	Male	49	CSF	HIV(+)	5	A	α
JXC283	Shangrao	Male	26	CSF	HIV(+); Bacterial pneumonia; Leukopenia; Hypoproteinemia	5	A	α
JXC285	Shangrao	Female	45	Blood	HIV(+); Asthma with glucocorticoid therapy; Bronchitis	5	A	α
JXC287	Ji'an	Female	62	Blood	Bacterial pneumonia	5	A	α
JXC288	Shangrao	Female	57	CSF	Diabetes(typeII); Hypertension	5	A	α
JXC289	Shangrao	Male	59	CSF	Leukopenia; Hypoproteinemia	5	A	α
JXC291	Yingtan	Male	49	CSF	HIV(+)	5	A	α
JXC292	Nanchang	Female	67	Hydrothorax	Unknown	5	A	α
JXC294	Nanchang	Female	55	CSF	Unknown	5	A	α
JXC296	Shangrao	Female	60	CSF	Hydrocephalus; Cerebral infarction	5	A	α
JXC298	Shangrao	Male	58	Blood	Unknown	5	A	α
JXC300	Shangrao	Female	57	CSF	Diabetes(typeII); Hypertension	5	A	α
JXC301	Nanchang	Male	26	Blood	HIV(+); Hemiplegia; Disseminated Talaromycosis	5	A	α
JXC302	Shangrao	Male	49	Blood	HIV(+)	5	A	α
JXC303	Shangrao	Female	50	CSF	HIV(+)	5	A	α

JXC304	Jiujiang	Male	55	CSF	HIV(+); Bacterial pneumonia	359	A	α

a:sequence type; b:mating type.
