# Supplementary material for: High Prevalence of HIV-Related Cryptococcosis and Increased Resistance to Fluconazole of the Cryptococcus neoformans Complex in Jiangxi Province, South Central China
Source: Front Cell Infect Microbiol. 2021 Nov 1;11:723251. doi: 10.3389/fcimb.2021.723251 (PMC8592285; doi:10.3389/fcimb.2021.723251)
Supplement: Supplementary Table 3 — Susceptibilities of the 86 C. neoformans isolates from Jiangxi Province against six common antifungal drugs. [file DataSheet_3.pdf]

Table S3. Susceptibilities of the 86 *C. neoformans* isolates from Jiangxi Province against six common antifungal drugs

Isolate	Location	ST[a]	FLU[b]	ITR[c]	AMB[d]	POS[e]	VOR[f]	5FC[g]
JXC003	Shangrao	6	16	0.25	1	0.5	0.25	1
JXC017	Yingtan	5	16	0.5	1	1	0.125	16
JXC019	Jiujiang	5	16	0.25	0.5	0.5	0.125	8
JXC034	Ji'an	359	16	0.5	1	0.5	0.125	4
JXC038	Shangrao	6	16	0.5	1	1	0.25	>64
JXC041	Yingtan	5	16	0.5	1	1	0.125	2
JXC047	Shangrao	6	16	0.5	0.5	0.5	0.125	0.5
JXC054	Shangrao	5	64	1	1	1	0.5	8
JXC055	Ji'an	5	16	0.5	1	1	0.125	2
JXC078	Yichun	5	32	0.5	1	0.5	0.125	2
JXC081	Yichun	5	16	0.25	1	0.5	0.125	16
JXC113	Nanchang	5	16	0.5	2	0.25	0.06	8
JXC125	Nanchang	5	16	0.5	1	0.5	0.125	4
JXC135	Nanchang	5	16	0.5	2	0.5	0.125	2
JXC136	Shangrao	359	32	0.06	4	0.06	0.06	0.125
JXC143	Shangrao	5	32	0.5	1	1	0.25	2
JXC144	Nanchang	5	16	0.25	1	1	0.125	1
JXC145	Shangrao	5	16	0.5	2	1	0.125	8
JXC179	Nanchang	5	16	0.5	1	0.5	0.125	4
JXC191	Yingtan	5	32	0.5	4	0.5	0.5	4
JXC192	Nanchang	5	16	0.5	1	0.5	0.125	4
JXC229	Nanchang	5	16	0.5	1	0.5	0.125	2
JXC001	Yingtan	5	8	0.5	1	0.5	0.25	4
JXC004	Ganzhou	5	8	0.5	1	0.25	0.125	2
JXC006	Shangrao	5	8	0.5	0.5	0.25	0.125	1
JXC008	Nanchang	5	8	0.5	1	0.25	0.125	8
JXC010	Nanchang	656	8	0.5	0.5	0.125	0.0625	1
JXC011	Jiujiang	5	8	0.5	1	0.5	0.125	4
JXC012	Ji'an	5	8	0.25	0.5	0.25	0.0625	1
JXC013	Ji'an	359	8	0.5	2	0.5	0.125	>64
JXC014	Yichun	5	8	0.5	1	0.5	0.125	2
JXC016	Ji'an	359	8	0.25	0.5	0.25	0.0625	>64
JXC018	Shangrao	5	8	0.25	0.5	0.5	0.125	1
JXC020	Jingdezhen	5	8	0.25	1	0.25	0.125	1
JXC022	Shangrao	657	8	0.25	1	0.5	0.125	1
JXC026	Jingdezhen	5	8	0.5	1	0.5	0.0625	2
JXC027	Jingdezhen	5	8	0.25	1	0.5	0.0625	1
JXC030	Yichun	81	8	0.25	1	0.5	0.125	2
JXC031	Yichun	5	8	0.25	1	0.5	0.125	2
JXC032	Yichun	658	8	0.25	1	0.5	0.125	>64
JXC033	Fuzhou	5	8	0.5	1	0.5	0.0625	1

JXC036	Xinyu	5	8	0.25	1	0.25	0.0625	1
JXC037	Jingdezhen	5	8	0.5	1	0.5	0.0625	1
JXC039	Nanchang	5	8	0.5	1	1	0.125	1
JXC042	Nanchang	5	8	0.5	0.5	1	0.125	0.5
JXC043	Nanchang	5	8	0.25	0.5	1	0.0625	4
JXC045	Ganzhou	5	8	0.25	0.5	0.25	0.125	1
JXC048	Nanchang	359	8	0.5	1	1	0.0625	2
JXC049	Nanchang	5	8	0.5	1	1	0.0625	2
JXC050	Nanchang	5	8	0.5	0.5	1	0.125	0.5
JXC051	Jingdezhen	5	8	0.25	1	0.5	0.0625	1
JXC056	Jiujiang	5	8	0.25	1	0.5	0.0625	2
JXC057	Nanchang	5	8	0.5	1	0.5	0.0625	1
JXC059	Yichun	5	8	0.5	1	1	0.0625	1
JXC060	Nanchang	5	8	0.5	1	1	0.0625	4
JXC065	Yichun	5	8	0.25	1	0.5	0.0625	1
JXC066	Jiujiang	5	8	0.25	0.5	0.25	0.0625	1
JXC068	Yingtan	5	8	0.5	0.5	0.5	0.0625	1
JXC070	Ji'an	5	8	0.25	0.5	0.0625	0.0625	>64
JXC071	Nanchang	5	8	0.5	1	1	0.125	0.5
JXC075	Shangrao	5	8	0.25	1	0.5	0.0625	1
JXC088	Yichun	5	8	0.5	1	0.5	0.125	1
JXC091	Shangrao	5	8	0.5	2	1	0.125	16
JXC095	Shangrao	5	8	0.5	1	1	0.125	2
JXC096	Shangrao	5	8	0.5	1	1	0.125	8
JXC102	Yichun	5	8	0.5	0.5	1	0.0625	2
JXC114	Shangrao	5	8	0.5	1	0.5	0.0625	2
JXC115	Nanchang	5	8	0.5	1	1	0.0625	2
JXC146	Shangrao	5	8	0.25	1	1	0.0625	16
JXC153	Shangrao	5	8	0.5	1	1	0.0625	2
JXC165	Jiujiang	5	8	0.5	1	1	0.0625	2
JXC167	Nanchang	5	8	0.25	0.5	1	0.0625	2
JXC177	Shangrao	5	8	0.5	1	1	0.0625	4
JXC181	Nanchang	5	8	0.5	1	1	0.0625	>64
JXC194	Ji'an	5	8	0.25	1	0.5	0.0625	1
JXC231	Yingtan	5	8	0.5	1	1	0.125	4
JXC234	Ji'an	5	8	0.5	1	1	0.125	2
JXC235	Shangrao	5	8	0.5	1	1	0.0625	1
JXC259	Jiujiang	5	8	0.25	1	1	0.0625	>64
JXC260	Jiujiang	5	8	0.25	1	0.5	0.0625	>64
JXC264	Yichun	5	8	0.5	1	1	0.25	2
JXC278	Jingdezhen	5	8	0.5	1	0.5	0.125	8
JXC279	Shangrao	5	8	0.5	1	1	0.125	4
JXC280	Shangrao	5	8	0.5	1	0.5	0.0625	1
JXC283	Shangrao	5	8	0.5	0.5	1	0.125	2

JXC304	Jiujiang	359	8	0.5	1	0.5	0.125	1

a: sequence type; b: fluconazole, μg/ml; c: itraconazole, μg/ml; d: amphotericin B, μg/ml; e:Posaconazole, μg/ml; f:voriconazole, μg/ml; g: 5-fluorocytosine, μg/ml.
