# Supplementary material for: High Prevalence of HIV-Related Cryptococcosis and Increased Resistance to Fluconazole of the Cryptococcus neoformans Complex in Jiangxi Province, South Central China
Source: Front Cell Infect Microbiol. 2021 Nov 1;11:723251. doi: 10.3389/fcimb.2021.723251 (PMC8592285; doi:10.3389/fcimb.2021.723251)
Supplement: Supplementary Table 4 — The list of gene bank accession numbers for seven genes of 199 isolates. [file DataSheet_4.pdf]

Table S4. The list of gene bank accession numbers for seven genes of 199 isolates

Isolate	Specimen	Gene Bank Accession No.						
		IGS1	CAP59	GPD1	LAC1	URA5	SOD1	PLB1
JXC001	CSF	MZ406629	MZ406828	MZ407027	MZ406032	MZ405833	MZ406430	MZ406231
JXC002	CSF	MZ406630	MZ406829	MZ407028	MZ406033	MZ405834	MZ406431	MZ406232
JXC003	CSF	MZ406631	MZ406830	MZ407029	MZ406034	MZ405835	MZ406432	MZ406233
JXC004	CSF	MZ406632	MZ406831	MZ407030	MZ406035	MZ405836	MZ406433	MZ406234
JXC005	Blood	MZ406633	MZ406832	MZ407031	MZ406036	MZ405837	MZ406434	MZ406235
JXC006	CSF	MZ406634	MZ406833	MZ407032	MZ406037	MZ405838	MZ406435	MZ406236
JXC008	Blood	MZ406635	MZ406834	MZ407033	MZ406038	MZ405839	MZ406436	MZ406237
JXC009	CSF	MZ406636	MZ406835	MZ407034	MZ406039	MZ405840	MZ406437	MZ406238
**JXC010**	CSF	MZ406637	MZ406836	MZ407035	MZ406040	MZ405841	**MZ406438**	MZ406239
JXC011	CSF	MZ406638	MZ406837	MZ407036	MZ406041	MZ405842	MZ406439	MZ406240
JXC012	CSF	MZ406639	MZ406838	MZ407037	MZ406042	MZ405843	MZ406440	MZ406241
JXC013	Blood	MZ406640	MZ406839	MZ407038	MZ406043	MZ405844	MZ406441	MZ406242
JXC014	Blood	MZ406641	MZ406840	MZ407039	MZ406044	MZ405845	MZ406442	MZ406243
JXC016	CSF	MZ406642	MZ406841	MZ407040	MZ406045	MZ405846	MZ406443	MZ406244
JXC017	CSF	MZ406643	MZ406842	MZ407041	MZ406046	MZ405847	MZ406444	MZ406245
JXC018	CSF	MZ406644	MZ406843	MZ407042	MZ406047	MZ405848	MZ406445	MZ406246
JXC019	CSF	MZ406645	MZ406844	MZ407043	MZ406048	MZ405849	MZ406446	MZ406247
JXC020	Blood	MZ406646	MZ406845	MZ407044	MZ406049	MZ405850	MZ406447	MZ406248
JXC021	CSF	MZ406647	MZ406846	MZ407045	MZ406050	MZ405851	MZ406448	MZ406249
**JXC022**	CSF	MZ406648	MZ406847	MZ407046	MZ406051	MZ405852	MZ406449	**MZ406250**
JXC023	CSF	MZ406649	MZ406848	MZ407047	MZ406052	MZ405853	MZ406450	MZ406251
JXC025	Blood	MZ406650	MZ406849	MZ407048	MZ406053	MZ405854	MZ406451	MZ406252
JXC026	Blood	MZ406651	MZ406850	MZ407049	MZ406054	MZ405855	MZ406452	MZ406253

JXC027	Blood	MZ406652	MZ406851	MZ407050	MZ406055	MZ405856	MZ406453	MZ406254
JXC028	CSF	MZ406653	MZ406852	MZ407051	MZ406056	MZ405857	MZ406454	MZ406255
JXC030	CSF	MZ406654	MZ406853	MZ407052	MZ406057	MZ405858	MZ406455	MZ406256
JXC031	CSF	MZ406655	MZ406854	MZ407053	MZ406058	MZ405859	MZ406456	MZ406257
**JXC032**	CSF	**MZ406656**	MZ406855	MZ407054	MZ406059	MZ405860	MZ406457	MZ406258
JXC033	CSF	MZ406657	MZ406856	MZ407055	MZ406060	MZ405861	MZ406458	MZ406259
JXC034	CSF	MZ406658	MZ406857	MZ407056	MZ406061	MZ405862	MZ406459	MZ406260
JXC035	Blood	MZ406659	MZ406858	MZ407057	MZ406062	MZ405863	MZ406460	MZ406261
JXC036	CSF	MZ406660	MZ406859	MZ407058	MZ406063	MZ405864	MZ406461	MZ406262
JXC037	Blood	MZ406661	MZ406860	MZ407059	MZ406064	MZ405865	MZ406462	MZ406263
JXC038	Blood	MZ406662	MZ406861	MZ407060	MZ406065	MZ405866	MZ406463	MZ406264
JXC039	CSF	MZ406663	MZ406862	MZ407061	MZ406066	MZ405867	MZ406464	MZ406265
JXC040	Blood	MZ406664	MZ406863	MZ407062	MZ406067	MZ405868	MZ406465	MZ406266
JXC041	CSF	MZ406665	MZ406864	MZ407063	MZ406068	MZ405869	MZ406466	MZ406267
JXC042	CSF	MZ406666	MZ406865	MZ407064	MZ406069	MZ405870	MZ406467	MZ406268
JXC043	Blood	MZ406667	MZ406866	MZ407065	MZ406070	MZ405871	MZ406468	MZ406269
JXC044	CSF	MZ406668	MZ406867	MZ407066	MZ406071	MZ405872	MZ406469	MZ406270
JXC045	CSF	MZ406669	MZ406868	MZ407067	MZ406072	MZ405873	MZ406470	MZ406271
JXC046	CSF	MZ406670	MZ406869	MZ407068	MZ406073	MZ405874	MZ406471	MZ406272
JXC047	Blood	MZ406671	MZ406870	MZ407069	MZ406074	MZ405875	MZ406472	MZ406273
JXC048	Blood	MZ406672	MZ406871	MZ407070	MZ406075	MZ405876	MZ406473	MZ406274
JXC049	Blood	MZ406673	MZ406872	MZ407071	MZ406076	MZ405877	MZ406474	MZ406275
JXC050	CSF	MZ406674	MZ406873	MZ407072	MZ406077	MZ405878	MZ406475	MZ406276
JXC051	CSF	MZ406675	MZ406874	MZ407073	MZ406078	MZ405879	MZ406476	MZ406277
JXC052	Blood	MZ406676	MZ406875	MZ407074	MZ406079	MZ405880	MZ406477	MZ406278
JXC054	Blood	MZ406677	MZ406876	MZ407075	MZ406080	MZ405881	MZ406478	MZ406279

JXC055	CSF	MZ406678	MZ406877	MZ407076	MZ406081	MZ405882	MZ406479	MZ406280
JXC056	Blood	MZ406679	MZ406878	MZ407077	MZ406082	MZ405883	MZ406480	MZ406281
JXC057	CSF	MZ406680	MZ406879	MZ407078	MZ406083	MZ405884	MZ406481	MZ406282
JXC058	Blood	MZ406681	MZ406880	MZ407079	MZ406084	MZ405885	MZ406482	MZ406283
JXC059	CSF	MZ406682	MZ406881	MZ407080	MZ406085	MZ405886	MZ406483	MZ406284
JXC060	CSF	MZ406683	MZ406882	MZ407081	MZ406086	MZ405887	MZ406484	MZ406285
JXC061	Blood	MZ406684	MZ406883	MZ407082	MZ406087	MZ405888	MZ406485	MZ406286
JXC062	CSF	MZ406685	MZ406884	MZ407083	MZ406088	MZ405889	MZ406486	MZ406287
JXC065	Blood	MZ406686	MZ406885	MZ407084	MZ406089	MZ405890	MZ406487	MZ406288
JXC066	CSF	MZ406687	MZ406886	MZ407085	MZ406090	MZ405891	MZ406488	MZ406289
JXC068	CSF	MZ406688	MZ406887	MZ407086	MZ406091	MZ405892	MZ406489	MZ406290
JXC070	CSF	MZ406689	MZ406888	MZ407087	MZ406092	MZ405893	MZ406490	MZ406291
JXC071	Blood	MZ406690	MZ406889	MZ407088	MZ406093	MZ405894	MZ406491	MZ406292
JXC075	Blood	MZ406691	MZ406890	MZ407089	MZ406094	MZ405895	MZ406492	MZ406293
JXC078	CSF	MZ406692	MZ406891	MZ407090	MZ406095	MZ405896	MZ406493	MZ406294
JXC081	CSF	MZ406693	MZ406892	MZ407091	MZ406096	MZ405897	MZ406494	MZ406295
JXC084	Blood	MZ406694	MZ406893	MZ407092	MZ406097	MZ405898	MZ406495	MZ406296
JXC087	CSF	MZ406695	MZ406894	MZ407093	MZ406098	MZ405899	MZ406496	MZ406297
JXC088	CSF	MZ406696	MZ406895	MZ407094	MZ406099	MZ405900	MZ406497	MZ406298
JXC091	CSF	MZ406697	MZ406896	MZ407095	MZ406100	MZ405901	MZ406498	MZ406299
JXC092	Blood	MZ406698	MZ406897	MZ407096	MZ406101	MZ405902	MZ406499	MZ406300
JXC095	Blood	MZ406699	MZ406898	MZ407097	MZ406102	MZ405903	MZ406500	MZ406301
JXC096	Blood	MZ406700	MZ406899	MZ407098	MZ406103	MZ405904	MZ406501	MZ406302
JXC097	Blood	MZ406701	MZ406900	MZ407099	MZ406104	MZ405905	MZ406502	MZ406303
JXC098	Blood	MZ406702	MZ406901	MZ407100	MZ406105	MZ405906	MZ406503	MZ406304
JXC101	Blood	MZ406703	MZ406902	MZ407101	MZ406106	MZ405907	MZ406504	MZ406305

JXC102	Blood	MZ406704	MZ406903	MZ407102	MZ406107	MZ405908	MZ406505	MZ406306
JXC104	CSF	MZ406705	MZ406904	MZ407103	MZ406108	MZ405909	MZ406506	MZ406307
JXC105	CSF	MZ406706	MZ406905	MZ407104	MZ406109	MZ405910	MZ406507	MZ406308
JXC107	CSF	MZ406707	MZ406906	MZ407105	MZ406110	MZ405911	MZ406508	MZ406309
JXC109	CSF	MZ406708	MZ406907	MZ407106	MZ406111	MZ405912	MZ406509	MZ406310
JXC110	CSF	MZ406709	MZ406908	MZ407107	MZ406112	MZ405913	MZ406510	MZ406311
JXC111	CSF	MZ406710	MZ406909	MZ407108	MZ406113	MZ405914	MZ406511	MZ406312
JXC113	Blood	MZ406711	MZ406910	MZ407109	MZ406114	MZ405915	MZ406512	MZ406313
JXC114	CSF	MZ406712	MZ406911	MZ407110	MZ406115	MZ405916	MZ406513	MZ406314
JXC115	CSF	MZ406713	MZ406912	MZ407111	MZ406116	MZ405917	MZ406514	MZ406315
JXC123	CSF	MZ406714	MZ406913	MZ407112	MZ406117	MZ405918	MZ406515	MZ406316
JXC124	CSF	MZ406715	MZ406914	MZ407113	MZ406118	MZ405919	MZ406516	MZ406317
JXC125	CSF	MZ406716	MZ406915	MZ407114	MZ406119	MZ405920	MZ406517	MZ406318
JXC126	CSF	MZ406717	MZ406916	MZ407115	MZ406120	MZ405921	MZ406518	MZ406319
JXC129	CSF	MZ406718	MZ406917	MZ407116	MZ406121	MZ405922	MZ406519	MZ406320
JXC130	Blood	MZ406719	MZ406918	MZ407117	MZ406122	MZ405923	MZ406520	MZ406321
JXC132	Blood	MZ406720	MZ406919	MZ407118	MZ406123	MZ405924	MZ406521	MZ406322
JXC133	Blood	MZ406721	MZ406920	MZ407119	MZ406124	MZ405925	MZ406522	MZ406323
JXC134	Blood	MZ406722	MZ406921	MZ407120	MZ406125	MZ405926	MZ406523	MZ406324
JXC135	CSF	MZ406723	MZ406922	MZ407121	MZ406126	MZ405927	MZ406524	MZ406325
JXC136	Blood	MZ406724	MZ406923	MZ407122	MZ406127	MZ405928	MZ406525	MZ406326
JXC138	CSF	MZ406725	MZ406924	MZ407123	MZ406128	MZ405929	MZ406526	MZ406327
JXC140	CSF	MZ406726	MZ406925	MZ407124	MZ406129	MZ405930	MZ406527	MZ406328
JXC141	CSF	MZ406727	MZ406926	MZ407125	MZ406130	MZ405931	MZ406528	MZ406329
JXC143	CSF	MZ406728	MZ406927	MZ407126	MZ406131	MZ405932	MZ406529	MZ406330
JXC144	Blood	MZ406729	MZ406928	MZ407127	MZ406132	MZ405933	MZ406530	MZ406331

JXC145	CSF	MZ406730	MZ406929	MZ407128	MZ406133	MZ405934	MZ406531	MZ406332
JXC146	Blood	MZ406731	MZ406930	MZ407129	MZ406134	MZ405935	MZ406532	MZ406333
JXC147	CSF	MZ406732	MZ406931	MZ407130	MZ406135	MZ405936	MZ406533	MZ406334
JXC148	CSF	MZ406733	MZ406932	MZ407131	MZ406136	MZ405937	MZ406534	MZ406335
JXC149	CSF	MZ406734	MZ406933	MZ407132	MZ406137	MZ405938	MZ406535	MZ406336
JXC151	Blood	MZ406735	MZ406934	MZ407133	MZ406138	MZ405939	MZ406536	MZ406337
JXC153	CSF	MZ406736	MZ406935	MZ407134	MZ406139	MZ405940	MZ406537	MZ406338
JXC154	CSF	MZ406737	MZ406936	MZ407135	MZ406140	MZ405941	MZ406538	MZ406339
JXC155	CSF	MZ406738	MZ406937	MZ407136	MZ406141	MZ405942	MZ406539	MZ406340
JXC157	CSF	MZ406739	MZ406938	MZ407137	MZ406142	MZ405943	MZ406540	MZ406341
JXC160	CSF	MZ406740	MZ406939	MZ407138	MZ406143	MZ405944	MZ406541	MZ406342
JXC161	CSF	MZ406741	MZ406940	MZ407139	MZ406144	MZ405945	MZ406542	MZ406343
JXC163	CSF	MZ406742	MZ406941	MZ407140	MZ406145	MZ405946	MZ406543	MZ406344
JXC164	CSF	MZ406743	MZ406942	MZ407141	MZ406146	MZ405947	MZ406544	MZ406345
JXC165	CSF	MZ406744	MZ406943	MZ407142	MZ406147	MZ405948	MZ406545	MZ406346
JXC167	CSF	MZ406745	MZ406944	MZ407143	MZ406148	MZ405949	MZ406546	MZ406347
JXC169	CSF	MZ406746	MZ406945	MZ407144	MZ406149	MZ405950	MZ406547	MZ406348
JXC170	CSF	MZ406747	MZ406946	MZ407145	MZ406150	MZ405951	MZ406548	MZ406349
JXC171	CSF	MZ406748	MZ406947	MZ407146	MZ406151	MZ405952	MZ406549	MZ406350
**JXC172**	CSF	**MZ406749**	MZ406948	MZ407147	MZ406152	MZ405953	MZ406550	MZ406351
JXC173	CSF	MZ406750	MZ406949	MZ407148	MZ406153	MZ405954	MZ406551	MZ406352
JXC174	Blood	MZ406751	MZ406950	MZ407149	MZ406154	MZ405955	MZ406552	MZ406353
JXC176	CSF	MZ406752	MZ406951	MZ407150	MZ406155	MZ405956	MZ406553	MZ406354
JXC177	CSF	MZ406753	MZ406952	MZ407151	MZ406156	MZ405957	MZ406554	MZ406355
JXC178	Blood	MZ406754	MZ406953	MZ407152	MZ406157	MZ405958	MZ406555	MZ406356
JXC179	Blood	MZ406755	MZ406954	MZ407153	MZ406158	MZ405959	MZ406556	MZ406357

JXC180	CSF	MZ406756	MZ406955	MZ407154	MZ406159	MZ405960	MZ406557	MZ406358
JXC181	CSF	MZ406757	MZ406956	MZ407155	MZ406160	MZ405961	MZ406558	MZ406359
JXC183	CSF	MZ406758	MZ406957	MZ407156	MZ406161	MZ405962	MZ406559	MZ406360
JXC184	CSF	MZ406759	MZ406958	MZ407157	MZ406162	MZ405963	MZ406560	MZ406361
JXC187	CSF	MZ406760	MZ406959	MZ407158	MZ406163	MZ405964	MZ406561	MZ406362
JXC188	CSF	MZ406761	MZ406960	MZ407159	MZ406164	MZ405965	MZ406562	MZ406363
JXC189	CSF	MZ406762	MZ406961	MZ407160	MZ406165	MZ405966	MZ406563	MZ406364
JXC191	CSF	MZ406763	MZ406962	MZ407161	MZ406166	MZ405967	MZ406564	MZ406365
JXC192	Blood	MZ406764	MZ406963	MZ407162	MZ406167	MZ405968	MZ406565	MZ406366
JXC194	Blood	MZ406765	MZ406964	MZ407163	MZ406168	MZ405969	MZ406566	MZ406367
JXC222	CSF	MZ406766	MZ406965	MZ407164	MZ406169	MZ405970	MZ406567	MZ406368
JXC225	CSF	MZ406767	MZ406966	MZ407165	MZ406170	MZ405971	MZ406568	MZ406369
JXC226	CSF	MZ406768	MZ406967	MZ407166	MZ406171	MZ405972	MZ406569	MZ406370
JXC227	CSF	MZ406769	MZ406968	MZ407167	MZ406172	MZ405973	MZ406570	MZ406371
JXC229	CSF	MZ406770	MZ406969	MZ407168	MZ406173	MZ405974	MZ406571	MZ406372
JXC230	CSF	MZ406771	MZ406970	MZ407169	MZ406174	MZ405975	MZ406572	MZ406373
JXC231	CSF	MZ406772	MZ406971	MZ407170	MZ406175	MZ405976	MZ406573	MZ406374
JXC233	Blood	MZ406773	MZ406972	MZ407171	MZ406176	MZ405977	MZ406574	MZ406375
JXC234	CSF	MZ406774	MZ406973	MZ407172	MZ406177	MZ405978	MZ406575	MZ406376
JXC235	CSF	MZ406775	MZ406974	MZ407173	MZ406178	MZ406029	MZ406576	MZ406377
**JXC236**	Blood	MZ406776	MZ406975	MZ407174	MZ406179	MZ405979	MZ406577	**MZ406378**
JXC237	CSF	MZ406777	MZ406976	MZ407175	MZ406180	MZ405980	MZ406578	MZ406379
**JXC239**	Blood	MZ406778	MZ406977	MZ407176	MZ406181	MZ405981	MZ406579	**MZ406380**
JXC240	CSF	MZ406779	MZ406978	MZ407177	MZ406182	MZ405982	MZ406580	MZ406381
JXC241	CSF	MZ406780	MZ406979	MZ407178	MZ406183	MZ405983	MZ406581	MZ406382
JXC242	CSF	MZ406781	MZ406980	MZ407179	MZ406184	MZ405984	MZ406582	MZ406383

JXC243	CSF	MZ406782	MZ406981	MZ407180	MZ406185	MZ405985	MZ406583	MZ406384
JXC244	Bone marrow	MZ406783	MZ406982	MZ407181	MZ406186	MZ405986	MZ406584	MZ406385
JXC246	CSF	MZ406784	MZ406983	MZ407182	MZ406187	MZ405987	MZ406585	MZ406386
JXC247	CSF	MZ406785	MZ406984	MZ407183	MZ406188	MZ405988	MZ406586	MZ406387
JXC249	CSF	MZ406786	MZ406985	MZ407184	MZ406189	MZ405989	MZ406587	MZ406388
JXC250	CSF	MZ406787	MZ406986	MZ407185	MZ406190	MZ405990	MZ406588	MZ406389
JXC251	CSF	MZ406788	MZ406987	MZ407186	MZ406191	MZ405991	MZ406589	MZ406390
JXC252	CSF	MZ406789	MZ406988	MZ407187	MZ406192	MZ405992	MZ406590	MZ406391
JXC253	CSF	MZ406790	MZ406989	MZ407188	MZ406193	MZ405993	MZ406591	MZ406392
JXC254	CSF	MZ406791	MZ406990	MZ407189	MZ406194	MZ405994	MZ406592	MZ406393
JXC255	CSF	MZ406792	MZ406991	MZ407190	MZ406195	MZ405995	MZ406593	MZ406394
JXC256	CSF	MZ406793	MZ406992	MZ407191	MZ406196	MZ405996	MZ406594	MZ406395
JXC257	Blood	MZ406794	MZ406993	MZ407192	MZ406197	MZ405997	MZ406595	MZ406396
JXC258	CSF	MZ406795	MZ406994	MZ407193	MZ406198	MZ405998	MZ406596	MZ406397
JXC259	CSF	MZ406796	MZ406995	MZ407194	MZ406199	MZ405999	MZ406597	MZ406398
JXC260	CSF	MZ406797	MZ406996	MZ407195	MZ406200	MZ406000	MZ406598	MZ406399
JXC261	CSF	MZ406798	MZ406997	MZ407196	MZ406201	MZ406001	MZ406599	MZ406400
JXC262	CSF	MZ406799	MZ406998	MZ407197	MZ406202	MZ406002	MZ406600	MZ406401
JXC264	CSF	MZ406800	MZ406999	MZ407198	MZ406203	MZ406003	MZ406601	MZ406402
JXC265	CSF	MZ406801	MZ407000	MZ407199	MZ406204	MZ406004	MZ406602	MZ406403
JXC266	CSF	MZ406802	MZ407001	MZ407200	MZ406205	MZ406005	MZ406603	MZ406404
JXC269	Blood	MZ406803	MZ407002	MZ407202	MZ406206	MZ406031	MZ406604	MZ406405
JXC270	CSF	MZ406804	MZ407003	MZ407201	MZ406207	MZ406006	MZ406605	MZ406406
JXC274	CSF	MZ406805	MZ407004	MZ407203	MZ406208	MZ406007	MZ406606	MZ406407
JXC275	CSF	MZ406806	MZ407005	MZ407204	MZ406209	MZ406008	MZ406607	MZ406408
JXC276	CSF	MZ406807	MZ407006	MZ407205	MZ406210	MZ406009	MZ406608	MZ406409

JXC277	CSF	MZ406808	MZ407007	MZ407206	MZ406211	MZ406010	MZ406609	MZ406410
JXC278	CSF	MZ406809	MZ407008	MZ407207	MZ406212	MZ406011	MZ406610	MZ406411
JXC279	CSF	MZ406810	MZ407009	MZ407208	MZ406213	MZ406012	MZ406611	MZ406412
JXC280	CSF	MZ406811	MZ407010	MZ407209	MZ406214	MZ406013	MZ406612	MZ406413
JXC282	CSF	MZ406812	MZ407011	MZ407210	MZ406215	MZ406014	MZ406613	MZ406414
JXC283	CSF	MZ406813	MZ407012	MZ407211	MZ406216	MZ406015	MZ406614	MZ406415
JXC285	Blood	MZ406814	MZ407013	MZ407212	MZ406217	MZ406016	MZ406615	MZ406416
JXC287	Blood	MZ406815	MZ407014	MZ407213	MZ406218	MZ406017	MZ406616	MZ406417
JXC288	CSF	MZ406816	MZ407015	MZ407214	MZ406219	MZ406018	MZ406617	MZ406418
JXC289	CSF	MZ406817	MZ407016	MZ407215	MZ406220	MZ406019	MZ406618	MZ406419
JXC291	CSF	MZ406818	MZ407017	MZ407216	MZ406221	MZ406020	MZ406619	MZ406420
JXC292	Hydrothorax	MZ406819	MZ407018	MZ407217	MZ406222	MZ406021	MZ406620	MZ406421
JXC294	CSF	MZ406820	MZ407019	MZ407218	MZ406223	MZ406022	MZ406621	MZ406422
JXC296	CSF	MZ406821	MZ407020	MZ407219	MZ406224	MZ406023	MZ406622	MZ406423
JXC298	Blood	MZ406822	MZ407021	MZ407220	MZ406225	MZ406024	MZ406623	MZ406424
JXC300	CSF	MZ406823	MZ407022	MZ407221	MZ406226	MZ406025	MZ406624	MZ406425
JXC301	Blood	MZ406824	MZ407023	MZ407222	MZ406227	MZ406030	MZ406625	MZ406426
JXC302	Blood	MZ406825	MZ407024	MZ407223	MZ406228	MZ406026	MZ406626	MZ406427
JXC303	CSF	MZ406826	MZ407025	MZ407224	MZ406229	MZ406027	MZ406627	MZ406428
JXC304	CSF	MZ406827	MZ407026	MZ407225	MZ406230	MZ406028	MZ406628	MZ406429
